# Duration of Antimicrobial Therapy in Community Acquired Pneumonia: Less Is More

**DOI:** 10.1155/2014/759138

**Published:** 2014-01-21

**Authors:** Marilia Rita Pinzone, Bruno Cacopardo, Lilian Abbo, Giuseppe Nunnari

**Affiliations:** ^1^Division of Infectious Diseases, Department of Clinical and Molecular Biomedicine, University of Catania, Via Palermo 636, ARNAS Garibaldi Nesima, 95125 Catania, Italy; ^2^Division of Infectious Diseases, Department of Medicine, Miller School of Medicine, University of Miami, Miami, FL 33136, USA

## Abstract

Community acquired pneumonia (CAP) represents the most common cause of infection-related morbidity and mortality worldwide. Appropriate treatment of CAP is challenging and sometimes limited by the availability to obtain rapid and timely identification of the etiologic agent in order to initiate or deescalate the correct antimicrobial therapy. As a consequence, prescribers frequently select empiric antimicrobial therapy using clinical judgment, local patterns of antimicrobial resistance, and, sometimes, individual patient expectations. These issues may contribute to prolonged courses of inappropriate therapy. In this review, we discuss the evidence and recommendations from international guidelines for the management of CAP and the clinical trials that specifically addressed duration of antimicrobial therapy for CAP in adults. In randomized controlled trials comparing the clinical efficacy of a short-course antimicrobial regimen versus an extended-course regimen, no differences in terms of clinical success, bacterial eradication, adverse events, and mortality were observed. The use of biomarkers, such as procalcitonin, to guide the initiation and duration of antimicrobial therapy may reduce total antibiotic exposure and treatment duration, healthcare costs, and the risk of developing antimicrobial resistance. In clinical practice, antimicrobial stewardship interventions may improve the management of CAP and may help in reducing treatment duration. Sometimes “less is more” in CAP.

## 1. Introduction

Community acquired pneumonia (CAP) is one of the leading causes of morbidity and mortality worldwide [1–3]. The annual incidence of CAP ranges from 5 to 11 cases per 1000 adults and is known to vary markedly with age, being higher in the very young and elderly people. A broad range of pathogens, including bacteria, atypical agents, and viruses, may be responsible for CAP. *Streptococcus pneumoniae* (*S. pneumoniae*) is the most common bacterial pathogen causing CAP and may account for up to 50% of cases. Other common pathogens include *Haemophilus influenzae* (*H. influenzae*), *Moraxella catarrhalis*, *Mycoplasma pneumoniae*, *Chlamydophila pneumoniae*, *Legionella* spp., and influenza viruses [1–3].

Several professional organizations have developed guidelines to improve the diagnosis and management of CAP addressing selection and timing of antimicrobial therapy and transition from intravenous to oral therapy in hospitalized patients (Table 1) [4–6]. However, despite the wide range of recommendations, less data are available regarding appropriate duration of antimicrobial therapy and there are some discrepancies between the published guidelines. Even though a 7–14-day course of antimicrobial therapy is the “traditional” recommendation to treat CAP in clinical practice [5–7], the superiority of long-term regimens over short-term ones has not been demonstrated in randomized controlled trials.

In 2007, the Infectious Diseases Society of America (IDSA) and American Thoracic Society (ATS) published the consensus guidelines for the management of CAP in adults [4]: the recommendation was to treat patients with CAP for a minimum of 5 days (level I evidence, strong recommendation); patients should be afebrile for 48–72 hours and should have no more than one CAP-associated sign of clinical instability prior to therapy discontinuation (level II evidence, moderate recommendation). In presence of extrapulmonary infections, such as meningitis or endocarditis, or if the initial therapy was not effective against the isolated pathogen, a longer duration of therapy is suggested (level III evidence, weak recommendation) [4]. In a previously published evidence-based guideline, the ATS recommended a 7–10-day course of antibiotics for pneumococcal pneumonia and a 10–14-day antimicrobial treatment for “atypical” pathogens (level III evidence) [7]. However, the ATS guideline recognized the possibility to treat patients with CAP for 5–7 days when using agents such as azithromycin, which has an extended serum and tissue half-life [7].

The British Thoracic Society (BTS) guidelines recommended a 7-day course of antimicrobial therapy for community managed patients and patients admitted to hospital with low or moderately severe, uncomplicated pneumonia (guideline statement grade C, insufficient evidence) [5]; a longer course of antimicrobial therapy was suggested in case of severe pneumonia, which may be extended to 14–21 days according to clinical response and to microbiological data (for example if *Staphylococcus aureus* or Gram-negative enteric bacilli are suspected or confirmed to be the causative agent of CAP) (guideline statement grade C, insufficient evidence) [5]. The 2011 Joint Taskforce of the European Respiratory Society and European Society for Clinical Microbiology and Infectious Diseases (ERS/ESCMID) guidelines for the management of CAP in adults recommended to guide treatment duration based on response to biomarkers such as procalcitonin [6]; in any case, the duration of antimicrobial therapy should not exceed 8 days in responding patients, defined on the basis of clinical criteria, including body temperature and respiratory and haemodynamic parameters (guideline statement grade C2, insufficient evidence, resulting from one or more randomized controlled trials (RCTs) but not systematic review or meta-analysis) [6].

The definition of the optimal duration of antimicrobial therapy appears to be an important element in the management of CAP. If short-course regimens are demonstrated to be as effective as long-course ones, the decrease in treatment duration may have a significant effect on overall antibiotic consumption, thus favouring cost-savings and reducing the risk of adverse reactions and the selection of drug-resistant organisms [8, 9]. Optimal duration will also be dependent on the pharmacokinetic and pharmacodynamic profile of the antimicrobial agent in order to achieve an adequate drug concentration at the site of infection for the length of time required for bacterial killing [10].

The rational basis of short-term antimicrobial therapy comes from *in vitro* time-kill studies, demonstrating a significant reduction of bacterial load within 24 hours, when the appropriate antibiotic is chosen [11, 12]; additionally, for antimicrobial agents exhibiting concentration-dependent killing properties, the bactericidal effect is enhanced by reaching the most effective area under the concentration-time curve (AUC) to minimal inhibitory concentration (MIC) ratio [13]. In order to summarize the available evidence from published RCTs which have specifically addressed the issue of duration of therapy for CAP, we focused our search on studies comparing the efficacy and tolerability of the same drug, used in the same daily dosage, for different durations of treatment. We subsequently reviewed studies comparing short-course to long-course antimicrobial regimens for the treatment of CAP.

## 2. Same Antimicrobial Regimen, Same Dose, and Different Duration of Therapy

In patients with CAP, several RCTs have shown that short-term antimicrobial regimens may be as effective as long-term ones when patients were randomized to receive the same dose of the same drug for a different length of time (Table 2). In a randomized, double-blind, placebo-controlled noninferiority trial carried out in the Netherlands [14], the authors compared a 3-day and 8-day course of amoxicillin (1 g intravenous (IV) every 6 hours) in adult patients hospitalized with mild-to-moderate CAP (pneumonia severity index (PSI) score ≤110). Patients showing a substantial improvement after an initial 3-day treatment were randomized to 5 days of oral amoxicillin (750 mg every 8 hours) or placebo. A total of 186 patients were enrolled in the study, of which 119 were randomized at day 3. The treatment arms had similar baseline characteristics, except for number of smokers and symptoms at admission, which were more severe in the 3-day treatment group. The cure rates were similar at day 10 (93% for both groups) and day 28 (90% in the 3-day arm versus 88% in the 8-day arm); both groups had similar resolution of symptoms, radiological outcome, adverse events, and mean length of hospital stay.

A 5-day course of ceftriaxone (1 g IV once daily) was reported to be as effective as a 10-day course in a cohort of adult inpatients with CAP [15]. Analogously, Siegel et al. compared the efficacy of a 7- and 10-day course of antimicrobial therapy for inpatients with moderately severe CAP [16]: 52 veterans were first treated with 2 days of cefuroxime (750 mg IV every 8 hours) and then randomly assigned to receive 8 days or 5 days of oral therapy with cefuroxime axetil (500 mg every 12 hours). Clinical success rates were similar (90.9% versus 87.5%, resp.), with no recurrences at followup.

Telithromycin, the first member of the ketolide family, displays a spectrum of activity covering typical and atypical/intracellular respiratory tract pathogens, including resistant strains [17]. Its pharmacokinetics and tissue penetration permit once-a-day administration over a short duration [18, 19]. In a multicentre, randomized, double-blind, parallel-group phase III clinical trial, whose primary objective was to determine the equivalence in clinical efficacy between oral telithromycin (given at a dose of 800 mg once daily for 5 or 7 days) and oral clarithromycin (given at a dose of 500 mg twice daily for 10 days), Tellier et al. enrolled 575 adult non-ICU inpatients and outpatients with clinical and radiological findings consistent with CAP [20]. The investigators showed comparable clinical efficacy, bacterial eradication rates, and safety between the shorter-course telithromycin (5 days and 7 days) and longer-course clarithromycin (10 day) regimens. Furthermore, numerically higher compliance rates (92.0%) were observed in the 5-day telithromycin arm, when compared with the 7-day telithromycin arm (90.1%) and 10-day clarithromycin group (85.1%).

Gemifloxacin, a fluoroquinolone approved for the treatment of CAP, displays a concentration-dependent killing activity, which appears favorable to short-course, high-dose antimicrobial regimens [21]. File Jr. et al. tested the efficacy of 320 mg once daily gemifloxacin short-course therapy for the outpatient treatment of mild-to-moderate CAP [22]; the investigators compared a 5-day and a 7-day regimen in a multicentre, double-blind, randomized study of 510 adults, including those having known risk factors, such as a history of cardiac conditions (hypertension, ischaemic heart disease, and congestive heart failure) or other diseases known to adversely affect pneumonia outcome (e.g., diabetes) [22]. No difference in clinical cure rates was identified; in fact, the clinical resolution at the end of therapy was 96% for both regimens and was similar at followup (95% versus 92% in the 5-day and 7-day arm, resp.). *S. pneumoniae *was the most common pathogen isolated and had 100% bacterial eradication from the 5-day treatment group, including multidrug-resistant strains. Bacterial response rates at the end of therapy were 94% and 96% for the 5-day and 7-day group, respectively, and 91% for both groups at follow-up. Both gemifloxacin regimens were well tolerated. Discontinuation of the study drug due to adverse events occurred infrequently: 1.2% in the 5-day cohort and 2% in the 7-day one. Elevation of alanine aminotransferase (ALT) and aspartate aminotransferase (AST) levels was the most common drug-related adverse event, with no difference between the two groups after adjusting for baseline levels. Diarrhea and rash were the only other adverse events occurring with a frequency of >2% in either cohort. Of note, the incidence of rash was lower in the 5-day arm (0.4% versus 2.8% in the 7-day group, *P* = 0.04); moreover, no serious treatment-related adverse events were reported in the 5-day group, in comparison with three serious treatment-related adverse events in the 7-day group, thus suggesting the potential greater tolerability of a shorter-course regimen. In another trial [23], a 750 mg dose of levofloxacin once daily for 5 days was found to be as effective as 500 mg for 10 days. However, in this study the effectiveness of short-course therapy alone could not be evaluated, because two different doses were used [23].

The aforementioned trials [14–16, 20, 22] were all included in a recent meta-analysis of Dimopoulos et al. [24]. The authors compared short- (≤7 days) versus long- (≥2 days difference) course monotherapy regimens for CAP, confirming that short-course antimicrobial therapy was equivalent to standard length of therapy for clinical cure and bacterial eradication rates, relapses, adverse events, and mortality. However, it remains to be established if these findings may be extended to combination regimens; furthermore, there is need of clinical trials enrolling patients with severe CAP, in order to evaluate the efficacy and tolerability of short-term antimicrobial therapy in this specific context.

## 3. Short-Course versus Long-Course Antimicrobial Regimens

Azithromycin is one of the antimicrobials most commonly studied when comparing short- and long-courses of therapy in CAP [25–34], because of its long half-life and elevated pulmonary concentrations. Azithromycin concentrations in lung tissue have been shown to remain above the minimum inhibitory concentration (MIC) of the most important respiratory pathogens up to 4 days after the administration of a single 500 mg dose [35]. O'Doherty and Muller evaluated the efficacy and tolerability of a 3-day, once-daily course of azithromycin (500 mg/day), in comparison with clarithromycin (250 mg twice daily for 10 days), in the oral treatment of 203 adult outpatients with mild-to-moderate CAP [28]. Clinical success rates (94% versus 95%, resp.) were similar, suggesting equal effectiveness of both regimens. 97% of isolated pathogens were eradicated in the azithromycin arm, compared with 91% in the clarithromycin arm. Of importance, azithromycin, but not clarithromycin, was found to eradicate all the baseline *H. influenzae* infections, thus in keeping with previous studies, which had shown the superior *in vitro* activity against *H. influenzae* of azithromycin, in comparison with clarithromycin [28]. The incidence of treatment-related adverse events was similar in the two groups (14% azithromycin versus 13% clarithromycin). However, clarithromycin treatment resulted in two serious treatment-related adverse events, which caused premature treatment discontinuation. On the other hand, none of the patients in the azithromycin group discontinued therapy as a result of adverse events. In addition, liver function test abnormalities were detected more frequently in the clarithromycin treatment group (3% versus 1%, resp.), but this difference was not statistically significant (*P* = 0.621). Similar findings have been reported in other studies favouring shorter courses of azithromycin therapy [29, 34].

In 1990, Schonwald et al. compared a 5-day course of oral azithromycin (500 mg on day 1, 250 mg on days 2 to 5) and a 10-day course of oral erythromycin (500 mg once daily), for the treatment of 101 patients aged 12–80 years with atypical pneumonia [31]. There were no differences in clinical cure rates and azithromycin was better tolerated than erythromycin. In an open, randomized, multicenter study comparing the efficacy and safety of a 3-day course of azithromycin with a l0-day course of roxithromycin for the treatment of atypical pneumonia, 150 adult inpatients were randomized to receive either oral azithromycin (500 mg once daily for 3 days) or roxithromycin (150 mg twice daily for 10 days). Clinical cure rates (98.9% versus 94.3%, resp., *P* = 0.179) and adverse events (2.2% versus 5.7%, resp., *P* = 0.274) were equivalent [30]. Two studies have evaluated the use of a single dose of a microsphere formulation of azithromycin [32, 33]. In a phase III, multicenter, randomized, double-blind trial, Drehobl et al. compared the efficacy and safety of a single 2 g dose of azithromycin microspheres to that of an extended-release formulation of clarithromycin (1 g/day for 7 days) for the treatment of 501 adult outpatients with mild-to-moderate CAP [33]. Clinical cure rates were similar for the two arms: 92.6% for azithromycin microspheres and 94.7% for extended-release clarithromycin, respectively. Furthermore, the two regimens were equally effective in eradicating bacterial pathogens (91.8% versus 90.5%, resp.,). Both drugs were well tolerated. Of note, all azithromycin microsphere patients were fully compliant with active treatment, whereas 6% of clarithromycin-treated patients did not complete the entire 7-day course of therapy. In the future, compliance-related advantages of single-dose therapy with azithromycin microspheres is the potential use as directly observed therapy which may reduce the likelihood of treatment failures and the emergence of resistant pathogens.

D'Ignazio et al. performed a randomized, double-blind, noninferiority study comparing a 7-day course of levofloxacin (500 mg/day) to a single 2 g dose of azithromycin microspheres in 427 adults with mild-to-moderate CAP [32]. Clinical cure rates (89.7% versus 93.7%, resp.) and bacterial eradication rates (90.7% versus 92.3%, resp.) were equivalent. Treatment-related adverse events were reported in 19.9% of subjects receiving azithromycin and 12.3% of those receiving levofloxacin (*P* = 0.032). Most adverse events were mild-to-moderate in severity; diarrhea was the most common, occurring in 12.3% and 4.7% of azithromycin and levofloxacin patients, respectively (*P* = 0.0063). Adverse events did not significantly affect compliance rates, which were high in both groups (100% in the azithromycin arm versus 95.3% in the levofloxacin arm).

In a multicentre, randomized, double-blind, active controlled, parallel-group trial, Léophonte et al. compared the efficacy and safety of a 7-day course of gemifloxacin (320 mg once daily) to that of a 10-day course of amoxicillin/clavulanate (1 g/125 mg three times daily) for the treatment of CAP of suspected pneumococcal origin [36]. Based on the ATS guideline stratification [7], no more than 17% of patients in each treatment group were classified as having a severe risk of mortality from CAP. Over 91% of patients in each group were hospitalized at the time of randomization. Short-course gemifloxacin was shown to be at least as effective as long-course amoxicillin/clavulanate. Clinical cure rates for the gemifloxacin and amoxicillin/clavulanate groups were 95.3% and 90.3% at end of therapy and 88.7% and 87.6% at followup, respectively. Bacteriologic response rates were 96.3% in the gemifloxacin group and 91.8% in the amoxicillin/clavulanate group at end of therapy, 87.2% versus 89.1%, respectively, at followup. Of importance, when severity of CAP (mortality risk) or bacteremia at screening were considered, gemifloxacin was associated with a higher response rate than amoxicillin/clavulanate. In fact, patients at severe risk of mortality from CAP achieved success rates of 100% in the gemifloxacin group and 88% in the amoxicillin/clavulanate group; in bacteremic patients, clinical success rates were 100% in the gemifloxacin group and 91% in the amoxicillin/clavulanate group. Drug-related events were reported by 18.6% of patients in the gemifloxacin group and 22.9% of patients in the amoxicillin/clavulanate group. The most frequently reported adverse events (≥5% incidence) were insomnia, diarrhea and headache in the gemifloxacin group (11.4%, 8.4%, and 5.4%, resp.) and diarrhea, and insomnia in the amoxicillin/clavulanate group (13.1% and 5.2%, resp.). There were no statistically significant differences between the treatment groups for any adverse events with an incidence of ≥5%. The proportion of discontinuations due to adverse events was lower in the gemifloxacin group (8.4%), in comparison to amoxicillin/clavulanate (9.8%).

In a meta-analysis of fifteen randomized trials, Li et al. compared short-course (7 days or less) to long-course (more than 7 day) therapy for the treatment of mild-to-moderate CAP [37]. Even though 4 of the antibiotic classes most commonly used for CAP (macrolide, fluoroquinolone, beta-lactam, and ketolide) were represented in these trials, most of them addressed azithromycin short-term use. No difference in terms of clinical success, bacterial eradication, adverse events, and mortality were found.

These results confirm that shorter courses of therapy for CAP may be as effective as longer ones (Table 3); in addition, short-term therapy may improve patient compliance, decrease adverse effects, and minimize the emergence of bacterial resistance. In clinical practice, antimicrobial stewardship interventions may improve the management of CAP and may help reducing treatment duration [38].

## 4. Procalcitonin Guidance of Antimicrobial Therapy in CAP

In recent years, procalcitonin (PCT) has emerged as a useful diagnostic and prognostic biomarker in bacterial infections [39, 40]. Several studies have proposed PCT-based algorithms to guide the initiation and duration of antimicrobial therapy in patients with CAP [41–44]. In an RCT of 172 low-risk outpatients with CAP, Long et al. randomized patients to receive a PCT-guided or standard antimicrobial therapy [44]. In the control group, antimicrobial treatment was based on current guidelines. Initiation of antimicrobial treatment in the PCT group was based on an algorithm using PCT serum levels [43]. PCT values correlated with the severity of CAP, as assessed by the PSI. Prescription of antibiotics on admission (84.4% in the procalcitonin guided group versus 97.5% control, *P* = 0.004) and total antibiotic exposure (relative risk (RR) 0.55, 95% confidence interval (CI): 0.51–0.60, *P* = 0.003) were reduced in the PCT group, in comparison with the control arm. Furthermore, median duration of antimicrobial treatment was two days shorter in the PCT arm (5 days, IQR 3–6) than in the control group (7 days, IQR 5–9, *P* < 0.001). Clinical, laboratory, and radiological outcomes were similar in the two groups at 4-week followup.

Christ-Crain et al. reported similar results in an open intervention trial involving 302 patients with all severities of CAP admitted to the emergency department [43]. In 15% of the patients in the PCT group and in 1% in the control group antibiotics were withheld on admission (*P* < 0.001); PCT-guidance reduced total antibiotic exposure (RR 0.52; 95% CI 0.48–0.55, *P* < 0.001) and antimicrobial treatment duration (median 5 days versus 12 days, *P* < 0.001). Clinical outcome at followup was similar in both groups. Of note, only the PCT group patients with a high PSI score (classes IV and V) had a significantly longer duration of treatment, in comparison to patients with a low PSI score (classes I to III). Analogously, only in the PCT group the mean duration of antimicrobial treatment was significantly longer in patients with positive blood cultures, compared with patients with negative cultures. On admission, patients who died during the course of the study had significantly higher levels of PCT, as compared with patients who survived (0.7 *μ*g/L (interquartile range (IQR) 0.4–3) versus 0.45 (IQR 0.2–2), *P* = 0.02); on the contrary, c-reactive protein (CRP) levels were similar, thus, suggesting that PCT may be a more reliable prognostic marker than CRP.

In a recent individual patient data meta-analysis of fourteen randomized trials, Schuetz et al. evaluated the impact of PCT-based therapeutic strategy to reduce antibiotic use in hospitalized patients with acute respiratory tract infections (ARI) [45]. The authors reported PCT-guided algorithms to be associated with reduced antibiotic exposure across different clinical settings and ARI diagnosis. Overall, no differences in mortality rates and treatment failure were found. These results were confirmed when analyzing the subgroup of patients with CAP: there was no difference in mortality in the PCT group compared to controls (9.2% versus 10.8%, adjusted odds ratio (aOR 0.89 (95% CI 0.64–1.23)), whereas the risk for treatment failure was lower in the PCT group (19.1% versus 23.4%, aOR 0.77 (95% CI 0.62–0.96), *P* = 0.02) as well as duration of antimicrobial therapy (median 7 versus 10 days; adjusted difference in days −3.34, 95% CI −3.79 to −2.88, *P* < 0.0001).

## 5. Conclusions

There are some important clinical messages that can be drawn from this review of studies addressing the issue of duration of antimicrobial therapy for CAP. First, short-course regimens may be as effective as long-course ones for the treatment of CAP. Secondly, the use of PCT embedded in clinical algorithms may have a significant clinical and public health impact to reduce antibiotic exposure, healthcare costs, and the risk of developing antimicrobial resistance. Global antimicrobial stewardship efforts should focus on appropriate duration of antimicrobial therapy. Sometimes “less is more” in CAP.

## Figures and Tables

**Table 1:** This table presents a summary of guidelines recommendations for the duration of antimicrobial treatment of CAP.

Guideline	Recommended duration	Grade/level of evidence
IDSA/ATS (2007) [4]	Patients with CAP should be treated for a minimum of 5 days (level I evidence*), should be afebrile for 48–72 h, and should have no more than 1 CAP-associated sign of clinical instability before discontinuation of therapy (level II evidence*). A longer duration of therapy may be needed if initial therapy was not active against the identified pathogen or if it was complicated by extrapulmonary infection, such as meningitis or endocarditis (level III evidence*).	Level I: high Level II: moderate Level III: low
ERS/ESCMID (2011) [6]	The duration of treatment should generally not exceed 8 days in a responding patient [C2]. Biomarkers, particularly PCT, may guide shorter treatment duration.	C2: Insufficient evidence, from 1 RCT or >1 RCT, but no systematic review or meta-analysis
BTS (2009) [5]	For community managed and for most patients admitted to hospital with low or moderate severity and uncomplicated pneumonia, 7 days of appropriate antibiotics is recommended. For those with high severity microbiologically undefined pneumonia, 7–10 days of treatment is proposed. This may need to be extended to 14 or 21 days according to clinical judgment, for example, where *S*. *aureus* or Gram-negative enteric bacilli pneumonia is suspected or confirmed. [C]	C: Formal combination of expert views

*Level I evidence: evidence from well-conducted, randomized controlled trials; level II evidence: evidence from well-designed, controlled trials without randomization (including cohort, patient series, and case-control studies); level III evidence: evidence from case studies and expert opinion.

ATS: American Thoracic Society; BTS: British Thoracic Society; CAP: community acquired pneumonia; ERS/ESCMID: European Respiratory Society and European Society for Clinical Microbiology and Infectious Diseases; IDSA: Infectious Diseases Society of America; PCT: procalcitonin; RCT: randomized controlled trial.

**Table 2:** Studies comparing the efficacy of short-course versus long-course antimicrobial regimens for the treatment of CAP, using the same dose of the same drug for a different length of time.

Study	*N*	Population	Short-course regimen*	Long-course regimen*
Siegel et al. 1999 [16]	52	Adult inpatients	Cefuroxime 750 mg IV every 8 h for 2 d, then cefuroxime axetil 500 mg every 12 h PO for 5 d	Cefuroxime 750 mg IV every 8 h for 2 d, then cefuroxime axetil 500 mg every 12 h PO for 8 d
Léophonte et al. 2002 [15]	244	Adult inpatients	Ceftriaxone 1 g IV once daily for 5 d	Ceftriaxone 1 g IV once daily for 10 d
Tellier et al. 2004 [20]	388	Adult inpatients and outpatients	Telithromycin 800 mg PO once daily for 5 d	Telithromycin 800 mg PO once daily for 7 d
El Moussaoui et al. 2006 [14]	119	Adult inpatients	Amoxicillin 1 g IV every 6 h for 3 d	Amoxicillin 1 g IV every 6 h for 3 d, then amoxicillin 750 mg PO every 8 h for 5 d
File Jr. et al. 2007 [22]	510	Adult outpatients	Gemifloxacin 320 mg PO once daily for 5 d	Gemifloxacin 320 mg PO once daily for 7 d

*No statistically significant differences in cure rates.

**Table 3:** Studies comparing the efficacy of short-course versus long-course antibiotic regimens for the treatment of CAP, using different antimicrobial agents.

Study	*N*	Population	Short-course regimen*	Long-course regimen*
Schonwald et al. 1990 [31]	101	Adult inpatients and outpatients	Azithromycin PO 500 mg on day 1, 250 mg on days 2 to 5	Erythromycin 500 mg PO once daily for 10 d
Bohte et al. 1995 [25]	40	Adult inpatients	Azithromycin PO 500 mg twice daily on day 1, once daily on days 2 to 5	Erythromycin 500 mg PO once daily for 10 d
Schonwald et al. 1994 [30]	150	Adult inpatients	Azithromycin 500 mg PO once daily for 3 d	Roxithromycin 150 mg PO twice daily for 10 d
Rizzato et al. 1995 [29]	40	Adult inpatients	Azithromycin 500 mg PO once daily for 3 d	Clarithromycin 250 mg PO twice daily for at least 8 d
O'Doherty and Muller 1998[28]	203	Adult outpatients	Azithromycin 500 mg PO once daily for 3 d	Clarithromycin 250 mg PO twice daily for 10 d
D'Ignazio et al. 2005 [32]	427	Adult outpatients	Azithromycin microspheres, a single 2 g dose PO	Levofloxacin 500 mg PO once daily for 7 d
Drehobl et al. 2005 [33]	501	Adult outpatients	Azithromycin microspheres, a single 2 g dose PO	Clarithromycin (extended-release formulation) 1 g PO once daily for 7 d
Léophonte et al. 2004 [36]	324	Adult inpatients and outpatients	Gemifloxacin 320 mg PO once daily for 7 d	Amoxicillin/clavulanate 1 g/125 mg PO three times daily for 10 d
Tellier et al. 2004 [20]	575	Adult inpatients and outpatients	Telithromycin 800 mg PO once daily for 5 d or 7 d	Clarithromycin 500 mg PO twice daily for 10 d

*No statistically significant differences in cure rates.
